# Non PCR-amplified Transcripts and AFLP fragments as reduced representations of the quail genome for 454 Titanium sequencing

**DOI:** 10.1186/1756-0500-3-214

**Published:** 2010-07-28

**Authors:** Sophie Leroux, Katia Feve, Florence Vignoles, Olivier Bouchez, Christophe Klopp, Céline Noirot, David Gourichon, Sabine Richard, Christine Leterrier, Catherine Beaumont, Francis Minvielle, Alain Vignal, Frédérique Pitel

**Affiliations:** 1 UMR INRA/ENVT Laboratoire de Génétique Cellulaire, INRA, 31326 Castanet-Tolosan, France; 2 Plateforme génomique (PlaGe), Génopole Toulouse-Midi-Pyrénées, INRA, 31326 Castanet-Tolosan, France; 3 Sigenae UR875 Biométrie et Intelligence Artificielle, INRA, 31326 Castanet-Tolosan, France; 4 Plateforme bioinformatique Genotoul UR875 Biométrie et Intelligence Artificielle, INRA, 31326 Castanet-Tolosan, France; 5 PEAT, Pôle d'Expérimentation Animale de Tours, INRA, 37380 Nouzilly, France; 6 Institut de Génomique Fonctionnelle de Lyon, ENS Lyon, 69364 Lyon, France; 7 UMR6175 Physiologie de la Reproduction et des Comportements, INRA, 37380 Nouzilly, France; 8 UR83 Recherche Avicoles, INRA, 37380 Nouzilly, France; 9 UMR 1313 INRA/AgroParisTech, Génétique animale et biologie intégrative GABI, 78352 Jouy-en-Josas, France

## Abstract

**Background:**

SNP (Single Nucleotide Polymorphism) discovery is now routinely performed using high-throughput sequencing of reduced representation libraries. Our objective was to adapt 454 GS FLX based sequencing methodologies in order to obtain the largest possible dataset from two reduced representations libraries, produced by AFLP (Amplified Fragment Length Polymorphism) for genomic DNA, and EST (Expressed Sequence Tag) for the transcribed fraction of the genome.

**Findings:**

The expressed fraction was obtained by preparing cDNA libraries without PCR amplification from quail embryo and brain. To optimize the information content for SNP analyses, libraries were prepared from individuals selected in three quail lines and each individual in the AFLP library was tagged. Sequencing runs produced 399,189 sequence reads from cDNA and 373,484 from genomic fragments, covering close to 250 Mb of sequence in total.

**Conclusions:**

Both methods used to obtain reduced representations for high-throughput sequencing were successful after several improvements.

The protocols may be used for several sequencing applications, such as *de novo *sequencing, tagged PCR fragments or long fragment sequencing of cDNA.

## Findings

Next-generation sequencing can now generate from hundreds to thousands of megabases worth of data at a time [1]. Although this is a great progress when compared to conventional Sanger sequencing, it remains costly to obtain SNP by whole-genome sequencing of many individuals, especially in species for which no assembly is available. Therefore, sequencing reduced representations libraries is still an efficient and sparing approach. We describe here two protocols used for high-throughput sequencing in quail with several improvements to existing methods [2,3]. These can be applied to 454 library preparations for several purposes, such as cDNA sequencing without sample amplification, genomic DNA sequencing of scarce samples, or genomic sample multiplexing.

### Transcripts library preparation

Total RNA was extracted from about 500 mg of adult quail brains (3 quail lines [4,5], 4 samples each) and total embryos (E8 stage, 3 quail lines, 2 samples each) according to the technique described by Le Meur et al [6], slightly modified. Briefly, tissues were homogenized with a tissue homogenizer (TH, OMNI International) with extraction solution (LiCl 3 M, urea 6 M, sodium acetate 10 mM, pH 5.6), and conserved overnight at 4°C. After centrifugation, pellets were washed twice with washing solution (LiCl 4 M, urea 8 M) and dissolved in 500 μl of TES (Tris 50 mM, EDTA 20 mM, SDS 0.5%, pH 7.5) with Proteinase K (100 μg/ml). After incubation (30 min, 37°C), total RNA was phenol extracted with Phase Lock Gel (Phase Lock Gel Heavy, 2 ml, 5 Prime) according to the manufacturer's instructions except for the use of LiCl 5 M instead of sodium acetate 2 M. Total RNA yield was estimated on a NanoDrop 1000 spectrophotometer (NanoDrop).

Next, a DNase treatment was performed to remove any potential remaining DNA. For 45 μg of total RNA per sample, 20 U of RNasine (Promega), 10 U of DNAse (Roche), 1× of PCR buffer (Invitrogen), and 1.5 mM of MgCl2 (Invitrogen) were added after which the samples were incubated 30 min at 37°C, before a purification step (RNeasy minElute Cleanup, Qiagen). A control PCR was systematically performed to check for the absence of genomic DNA. The quality of RNA samples was assessed using the Agilent 2100 Bioanalyzer (Agilent Technologies). Equivalent quantities of each sample were pooled to obtain 75 μg of treated total RNA, for each quail line.

Poly(A)^+ ^RNAs were then purified twice using the Dynabeads Oligo(dT)_25 _kit (Invitrogen). First strand cDNA was synthesized from the purified mRNA, using the Superscript II Reverse Transcriptase (Invitrogen), according to the manufacturer instructions, with a modified primer, GAGAGAGAGACTGGAG(T)_16_VN, containing the *Gsu*I restriction enzyme site (CTGGAGNNNNNNNNNNNNNNNN^, overhang NN-3'), to trim the polyA tail after cDNA synthesis. The other protocol modification was to use methylated dCTP (dm^5^CTP, Fermentas). The hemi-methylation is necessary to avoid internal cleavage by subsequent *Gsu*I digestion [7].

The second strand cDNA was synthesized according to the Second Strand Synthesis Invitrogen protocol by adding 1× of Second-Strand Reaction Buffer (Invitrogen), 0.2 mM of classical dNTP mix, 10 U of *E. coli *DNA Ligase (New England Biolabs), 40 U of *E. coli *DNA Polymerase (New England Biolabs), 2.5 U of *E. coli *RNase H (New England Biolabs). The samples were incubated 2 hours at 16°C. Ten units of T4 DNA polymerase (New England Biolabs) were added for 5 minutes and the reaction was stopped by adding EDTA to a final concentration of 30 mM. Samples were then incubated 15 min at 37°C with 5 U of Ribonuclease I (Fermentas). Next, cDNA were extracted by using phenol/chloroform and Phase Lock Gel (Phase Lock Gel Light, 2 ml, 5 Prime), followed by ethanol precipitation. The pellets were resuspended in 6 μl of water.

The double-strand cDNA was digested with 5 U of *Gsu*I for 60 minutes at 30°C, and the reaction was stopped by heating at 65°C for 20 minutes. The samples were then submitted to 454 sequencing, each library being prepared from the pooled cDNA obtained from two syntheses (namely issued from 2 × 75 μg of total RNA).

### AFLP fragments library preparation

Genomic DNA was extracted from blood samples of 6 animals (3 quail lines, 2 individuals each) with a rapid high-salt protocol [8]. AFLP fragments were generated as in [8], except that we only performed the preamplification step. Briefly, 400 ng of genomic DNA were digested with 10 U of *EcoR*I and *Taq*I restriction enzymes (New England Biolabs). Adaptors [8] were ligated to the DNA fragments obtained, and the ligation reaction was diluted 5-fold in water. PCR amplifications were carried out for each sample in 25 μl reactions containing 5 μl of diluted ligation, 0.2 units of Phusion high-fidelity DNA polymerase (New England Biolabs), 1× High-Fidelity buffer (New England Biolabs), 0.2 mM dNTP, and 0.2 μM of each primer on a GeneAmp PCR System 9700 thermocycler (Applied Biosystems).

PCR primers were modified from preamplification primers [8] (Table 1): to identify the animal-of-origin for each sequence, a 4-base tag was added to the 5'-end of each primer, each sample being amplified with a Taq- and an Eco- primers bearing the same tag. Primers were further modified by adding a 5'-phosphate to allow the ligation of the 454 sequencing primers. Unlike in the CRoPS™approach [2], we did not modify A and B sequencing primers and used the oligonucleotides delivered in the Roche kits.

**Table 1:** Primers used for AFLP amplification

**Primer Name**	**Sequence**
EcoApTACG	P-TACGCTGCGTTACCAATTCA
TaqApTACG	P-TACGGATGAGTCCTGACCGAA
EcoApTAGA	P-TAGACTGCGTTACCAATTCA
TaqApTAGA	P-TAGAGATGAGTCCTGACCGAA
EcoApTATC	P-TATCCTGCGTTACCAATTCA
TaqApTATC	P-TATCGATGAGTCCTGACCGAA
EcoApTCAG	P-TCAGCTGCGTTACCAATTCA
TaqApTCAG	P-TCAGGATGAGTCCTGACCGAA
EcoApTCGT	P-TCGTCTGCGTTACCAATTCA
TaqApTCGT	P-TCGTGATGAGTCCTGACCGAA
EcoApTCTA	P-TCTACTGCGTTACCAATTCA
TaqApTCTA	P-TCTAGATGAGTCCTGACCGAA
EcoApTGAT	P-TGATCTGCGTTACCAATTCA
TaqApTGAT	P-TGATGATGAGTCCTGACCGAA
EcoApTGCA	P-TGCACTGCGTTACCAATTCA
TaqApTGCA	P-TGCAGATGAGTCCTGACCGAA

The 5'-ends are phosphorylated. The first 4 bases are the identification tags.

Thirty-five PCR cycles were performed, each consisting of denaturation at 94°C for 30 s, annealing at 59°C for 30 s and elongation at 72°C for 60 s. The ramp rate was settled to 80%. Polishing of PCR products was realized by the addition of 0.6 U T4 DNA Polymerase (New England Biolabs), 1× BSA, 1× T4 buffer (New England Biolabs), 0.1 mM dNTP and incubation at 12°C for 15 min. EDTA (10 mM) was added to stop the enzymatic reaction (20 min incubation at 75°C). All 6 samples were pooled and loaded as a unique sample on a 1% agarose gel, electrophoresed in 1× TBE buffer, and visualized by staining with ethidium bromide. A 200-400 bp fraction was recovered from the gel by cutting out the corresponding band and purifying the DNA with the Nucleospin Extraction kit (NucleoSpin^® ^Extract II, Macherey-Nagel).

### 454 sequencing

Fragments were sequenced using the Roche 454 Life Sciences Genome Sequencer FLX following the manufacturer's instructions for the Titanium series (454 Life Science, Roche). Libraries were prepared according to the 454 protocol: nebulization (only for the cDNA), purification, and ligation of adaptors. The libraries were prepared with ~9 μg (AFLP fragments) or ~1 μg (cDNA fragments) using the "Titanium General Library Preparation Kit". Both genome representations were treated alike, except that AFLP fragments did not undergo the first 3 steps (nebulization, Ampure purification, fragment end polishing). Because the recovered quantities were very low, cDNA library preparation was optimized as in [9] by retrieving fragments through heat denaturation: samples were eluted in 45 μL water, vortexed, denatured for 2 min at 90°C, and transferred to ice. A pellet of beads was obtained with the magnet, and the supernatant was collected and mixed with TE 10/1 to a final concentration of 1×.

DNA fragments were amplified using the "GS FLX Titanium SV emPCR Kit" (cDNA) or "GS FLX Titanium LV emPCR Kit" (AFLP fragments). Sequencing on the Genome Sequencer FLX was performed using the "GS FLX Sequencing Kit Titanium Reagents XLR70".

### Sequence analyses

The sequences are available at NCBI (http://www.ncbi.nlm.nih.gov/, SRA database, Accession number SRP002189).

#### Genomic AFLP fragments

AFLP fragments were sequenced using a half-plate, producing 373,484 reads and nearly 92 Mb. The average depth (8.6× for 4,929 analysed contigs, but with 56% of sequences as singlets) could be improved by performing other sequencing runs. The average sequence length (246 bp) should be increased by a gel cut at a higher size around 400 bp, or a reamplification step with 2 to 3 selective nucleotides - to limit the number of different fragments and keep an acceptable coverage - followed by an Ampure purification to remove short fragments.

We tagged each sample individually before making the 454 library on the pooled individuals. This procedure can have many applications, by drastically reducing the cost time spent and the amount of samples needed for this step. Library preparation is an important step in the sequencing protocol, regarding both handling time and financial costs. Moreover, each library requires a minimum amount of DNA or RNA starting material. Here, a single library is prepared instead of one per sample when tagging and multiplexing individual libraries. In addition, when sample selection is performed by sizing on gel, multiplexing individuals before retrieving the DNA fraction decreases the number of gels cuts and DNA extractions before library preparation. An important point here is also to avoid potential bias in the representation of each individual genome that could be caused by unavoidable slight difference between gel migrations and size estimations when cutting the gels.

Out of 289,703 sequences longer than 120 bp, for which we analysed the presence of a tag, only a small proportion of sequences (454) did not show a tagged AFLP primer at either end, with a tagging efficiency of 99.99%. However, only 55.8% (161,478/289,249) of sequences bore a tag at both ends. Given the average length of the sequenced fragments (the fraction was obtained from a 200-400 bp gel cut, but fragment lengths spanned a larger, about 100-500 bp, interval) and the average sequence length (246 bp), one could have expected a larger proportion of double-tagged fragments. Ninety five percent (153,469) of the double-tagged sequences showed the same tag at both ends. The remaining 5% either were chimeras with two different tags or included 1 or 2 false tags due to sequencing errors (tags not present in the primers we used). The relative representation of each sample according to its identification by the tagging approach was variable, but acceptable. The most abundant tag was only twice as frequent as the least frequent one (table 2). As observed previously for the very 5'end of the tag [2,10], the presence of an AC sequence in the tag may have led to an underrepresentation of the TACG tag. Our experiments expand the use of CRoPS™[2] technology to the Titanium version of 454 sequencing, without the need for modified sequencing primers, and with a single preamplification step.

**Table 2:** Representation of each individual in the 153,469 double-tagged AFLP sequences

**Tag pair**	**Occurrences**	**Proportion (%)**
TACG	16418	10.7
TAGA	27842	18.1
TATC	34031	22.2
TCAG	25944	16.9
TCGT	28595	18.6
TCTA	20639	13.5

The occurrence number of each double-tag is shown.

Using AFLP fragments to obtain a reduced representation of the genome allows developing SNP markers even for species lacking a whole-genome draft sequence [2]. Furthermore, the amplification step allows using a very small amount of DNA for the library preparation. The major advantage of this method when compared to the gel cut RRL (Reduced Representation Library), described for example by Van Tassel in cattle [11], is the possibility to multiplex several individuals in the same library preparation by using tagged PCR-primers, which was first done by Binladen and co-workers for a mitochondrial PCR fragment [10]. The possibility offered here to identify the individual origin of each sequence and its SNP alleles allows to detect line-specific SNP, or to target directly for SNP informative in a dedicated cross, when sequencing F1 animals. These opportunities are absent for classical RRL sequencing without individual tagging. In addition, the use of 454 sequencing allowed the production of a sufficient amount of sequence flanking the SNP, which is essential for designing genotyping assays, in species where no genome draft is available [11]. This would not have been the case with the Illumina technology available at the time.

#### cDNA fragments

For cDNA fragment sequencing, 6 quarter-plate runs (one for each tissue in each line), with an additional 1/8 of run gave a total of 399,189 reads and 154.3 Mb of sequence in total. The heterogeneity of the results (from 37,385 to 134,598 sequences per quarter-plate run) was partially due to the variable efficiency of the library preparations and we expect improved yields in the future. As previously observed [12], 454 runs on cDNA produce a smaller quantity of sequences than runs on genomic DNA. These sequences were assembled into 31,010 contigs (average depth of 11, from 2 to 3049), 36,572 sequences remaining as singlets.

Our method brings two important improvements to the classical 454 cDNA library preparation protocol. First, through the use of the heat-denaturation step [9] instead of the melting one (single-strand DNA is released from the beads through NaOH denaturation), a greater proportion of fragments is retrieved, and the use of a PCR step in the cDNA preparation becomes useless. The higher yield of heat treatment is notably due to the breaking of the biotin-streptavidin interaction.

This can be of great benefit when only a small amount of RNA is available, and when one wishes to avoid additional possible bias associated with PCR amplification: as PCR amplification from complex mixtures may generate representational differences between fragments [13], we chose to avoid any PCR amplification prior to the library preparation for the cDNA experiment. This allows the observed difference between samples of sequence frequencies for a given gene, to exactly represent its level of differential expression. Second, the use of a modified oligodT primer for the Reverse-Transcription step, associated with cDNA hemimethylation and *Gsu*I enzyme digestion, as suggested by Shibata et al [7], dramatically decrease the loss of efficiency in 454 sequencing related to homopolymers, especially problematic in the case of cDNA due to the poly(T) tail [14]. In the present study, most of the 3'-end sequences were "polyA-cleaned" by the *Gsu*I treatment with only 647 fragments displaying the modified oligodT primer. A PCR-based method to limit the 3' homopolymer has also been described by Beldade [15], but would then have all the disadvantages related to PCR.

A preliminary analysis allowed detecting more than 8,500 putative SNP from these data: 6,888 from the cDNA sequences and 1,695 from the genomic fragments. Their experimental validation remains to be performed.

The methods presented in this paper may be used for several 454 sequencing applications like de *novo sequencing*, tagged PCR fragments sequencing or long fragment sequencing of cDNA.

## Competing interests

The authors declare that they have no competing interests.

## Authors' contributions

SL, KF, FV and OB carried out the molecular studies. CK, CN and FP performed the sequence analyses. DG, SRD, CA, CL, CB and FM carried out the animal design and experiments. FP and AV conceived the study, and participated in its design and coordination. FP, SL, KF and FV drafted the manuscript. All authors finalized and approved the manuscript.
